# Trauma occurs in social contexts

**DOI:** 10.3402/ejpt.v7.31389

**Published:** 2016-03-17

**Authors:** Marit Sijbrandij, Miranda Olff

**Affiliations:** 1 Department of Clinical Psychology, The Netherlands and EMGO Institute for Health and Care Research, VU University Amsterdam, NL-1081 HV Amsterdam, The Netherlands, Email: e.m.sijbrandij@vu.nl; 2 Department of Psychiatry, Academic Medical Center, University of Amsterdam, Amsterdam, The Netherlands Arq Psychotrauma Expert Group, Diemen, The Netherlands

Vilnius, Lithuania's capital, was the beautiful location of the 14th Conference of the European Society of Traumatic Stress studies (ESTSS) from June 10 to 13, 2015. The main theme of this two-yearly conference was “Trauma in changing societies: social contexts and clinical practice.” The topic of the conference is timely, unfortunately. The current crisis in Syria changed (and is still changing) societies, especially in the Middle East. Over half of the population of Syria has left their homes, of which over 4 million have fled to other countries for an unknown future (UNHCR, 2016; see also Hall & Olff, 2016; Purgato & Olff, 2015). Other recent events such as recent terrorist attacks in Europe have also had an impact on the social contexts in affected countries. Such circumstances are likely to have implications for clinical practice in psychotrauma. The idea that social context in which trauma occurs strongly affects the mental health impact of trauma has been acknowledged within ESTSS (Ajduković, 2013). The lack of social support after trauma is one of the most consistent predictors of PTSD (e.g., Ozer et al., 2003); the neurobiology underlying the beneficial effects of social support receives increasing attention (Koch et al., 2016a, 2016b; Olff, 2012; Olff et al., 2014) and research shows how the opposite, that is, social exclusion, may explain PTSD symptoms through tonic immobility (Mooren & Van Minnen, 2014). But social support is a complex construct and differences exist among countries in the perception of received and desired support (Zepinic, Bogic, & Priebe, 2012). This special issue presents the papers based on the keynote presentations and invited panel discussions during the meeting, and the majority of these papers are related to this overarching conference theme.

The first lines of the popular Lithuanian song “Trys milijonai” sound “Perhaps we fought for too long—and in too many wars. Perhaps too long we have been chanting Glory.” They refer to the many regional and world-wide conflicts in which Lithuania has been involved during the past century. The first paper of this special issue was written by Evaldas Kazlauskas and Pauline Želvienè from Vilnius University in Lithuania (Kazlauskas & Želvienè, 2016) and presents an overview of the emerging number of studies from the Baltic countries (Lithuania, Estonia, Latvia) on the psychological consequences of these events. Studies are presented on the effects of adverse events resulting from Russian occupation for 50 years until 1990, and on the effects of two major disasters that involved the Baltic populations (the Chernobyl Nuclear Power Station accident in 1986 and the sinking of the MS Estonia in 1994).

Stuart Turner (2015) focuses on the recent increase in refugees seeking asylum in Europe due to conflicts in Syria and Afghanistan, and implications that this “refugee crisis” may have on policy of refugee admissions and mental health care. Turner (2015) emphasises the importance of supporting economic growth and human rights in low- and middle-income countries, and of recognising the weaknesses of the policies and decision-making processes concerning asylum procedures for refugees.

Maercker and Hecker (2016) describe a social interpersonal framework model of posttraumatic stress disorder (PTSD), in which the role of affective reactions in response to others, relationships with close ones, and the broader culture and society such as the cultural values held on posttraumatic stress and resilience are outlined. Maercker and Hecker (2016) also provide implications for psychological trauma interventions addressing social affects, relationships, or communities.

The paper “Social attachments and traumatic stress” by Richard Bryant (2016) provides an overview of his exciting keynote lecture that—similar to the previous keynote—challenged the traditional individual focus on trauma and recovery in favour of a focus that includes also the larger social context in which trauma occurs. Bryant (2016) presents both experimental studies and large-scale social network analysis studies on social connections and attachments in relation to the recovery after traumatic experiences. Based on this review, Bryant (2016) argues that an individual's attachment style (either secure or avoidant) is likely to interact with the response to receiving social support, a notion that should be tested in further empirical studies.

In addition, three papers integrated invited panel discussions that were held at the conference. First, Magruder, Kassam-Adams, Thoresen, and Olff (2016) discuss and illustrate how a public health approach to trauma may inform research and prevention efforts. This is illustrated by an example of the response to the 2011 Utøya attacks in Norway (see also Thoresen, Aakvaag, Wentzel-Larsen, Dyb, & Hjemdal, 2012) by showing the policy responses and intervention efforts on the individual, community, and the societal levels.

Kazlauskas et al. (2016b) describe the current situation with respect to the availability of different treatment approaches for trauma-related symptoms in seven European countries: Georgia, Germany, Lithuania, the Netherlands, Poland, Switzerland, and Turkey. The countries appear to be very different in terms of awareness of health problems related to trauma, and whether evidence-based trauma treatments are available.

Finally, Thomaes et al. (2016) from the ESTSS Taskforce on Neurobiology provided a general overview of the main research challenges that trauma researchers in the field of neurobiology are facing. They provide suggestions towards enhancing the methodology in future studies of biological traces underlying the aetiology of PTSD, and pharmacological strategies to prevent or treat PTSD. This paper is also a call for more standardisation and greater European and international collaboration in this area.

With this special issue that presents highlights of the biennial psychotrauma conference in Europe with contributions from experts inside and outside Europe, we hope to boost future research on the role of the broader social context in which trauma occurs. This offers many possibilities for novel research topics and methods. To name a few, big data studies may examine the use of social media, and specific characteristics of communication between individuals early after mass trauma by tracking smartphone data. Studies may also apply frameworks and variables from related disciplines such as sociology or economics, for instance, human value orientations (cf. Maercker & Hecker, 2016) to study the development of trauma-related psychological distress within larger communities or to compare countries. We may need more research into social circumstances for PTSD treatment, like this study showing that EMDR may be effective in reducing PTSD and depression symptoms among Syrian refugees located in a refugee camp (Acarturk et al., 2015). An exciting new research area opens up, with many possibilities for collaboration between researchers in the field of trauma and beyond.

*Marit Sijbrandij* Department of Clinical Psychology The Netherlands and EMGO Institute for Health and Care Research VU University Amsterdam NL-1081 HV Amsterdam, The Netherlands Email: e.m.sijbrandij@vu.nl *Miranda Olff* Department of Psychiatry Academic Medical Center University of Amsterdam Amsterdam, The Netherlands Arq Psychotrauma Expert Group Diemen, The Netherlands
